# Engaging community health workers in maternal and newborn care in eastern Uganda

**DOI:** 10.3402/gha.v8.23968

**Published:** 2015-03-31

**Authors:** Monica Okuga, Margaret Kemigisa, Sarah Namutamba, Gertrude Namazzi, Peter Waiswa

**Affiliations:** 1 Department of Health Policy, Planning and Management, School of Public Health, Makerere University, Kampala, Uganda; 2 Private Consultant, Kampala, Uganda; 3 Global Health, Department of Public Health Sciences, Karolinska Institutet, Stockholm, Sweden; 4 Iganga-Mayuge Health Demographic Surveillance Site, Iganga-Mayuge, Uganda

**Keywords:** newborn health, maternal health, community health worker, pregnancy, postnatal care, Uganda

## Abstract

**Background:**

Community health workers (CHWs) have been employed in a number of low- and middle-income countries as part of primary health care strategies, but the packages vary across and even within countries. The experiences and motivations of a multipurpose CHW in providing maternal and newborn health have not been well described.

**Objective:**

This study examined the perceptions of community members and experiences of CHWs around promoting maternal and newborn care practices, and the self-identified factors that influence the performance of CHWs so as to inform future study design and programme implementation.

**Design:**

Data were collected using in-depth interviews with six local council leaders, ten health workers/CHW supervisors, and eight mothers. We conducted four focus group discussions with CHWs. Respondents included 14 urban and 18 rural CHWs. Key themes explored included the experience of CHWs according to their various roles, and the facilitators and barriers they encounter in their work particular to provision of maternal and newborn care. Qualitative data were analysed using manifest content analysis methods.

**Results:**

CHWs were highly appreciated in the community and seen as important contributors to maternal and newborn health at grassroots level. Factors that positively influence CHWs included being selected by and trained in the community; being trained in problem-solving skills; being deployed immediately after training with participation of local leaders; frequent supervision; and having a strengthened and responsive supply of services to which families can be referred. CHWs made use of social networks to identify pregnant and newly delivered women, and were able to target men and the wider family during health education activities. Intrinsic motivators (e.g. community appreciation and the prestige of being ‘a doctor’), monetary (such as a small transport allowance), and material incentives (e.g. bicycles, bags) were also important to varying degrees.

**Conclusions:**

There is a continued role for CHWs in improving maternal and newborn care and linking families with health services. However, the process for building CHW programmes needs to be adapted to the local setting, including the process of training, deployment, supervision, and motivation within the context of a responsive and available health system.

Globally 2.9 million babies die during their first month of life (the newborn period), and an additional 2.6 million babies are stillborn each year. About three-quarters of newborn deaths occur within the first week of life, with 25–45% occurring within the first 24 hours of life (1, 2). Technical agreement has advanced around what to do to improve health and survival for mothers and their babies in the poorest countries, but how to achieve these improvements remains a challenge. One approach has been the reinvigoration of comprehensive primary health care, including renewed recognition of the importance of community ownership and expanded use of health extension and community health workers (CHWs) (3).

The need for community-level access to care is highest in low-income countries. In Uganda, nearly all women (94%) access antenatal care (ANC) at least once, but deliveries at a health facility, while increasing, remain much lower (57%) (4). Only 59% of births are attended by a skilled health provider, with lower coverage in rural areas (4). One study, which looked at the location of neonatal deaths in eastern Uganda, revealed that 54% of newborns died outside of a health facility (5).

The feasibility of a community-based strategy in effectively reducing neonatal mortality within settings of weak health systems, low service utilisation, and high neonatal mortality has been demonstrated in trials and demonstration projects in Asia (6–9). In trials in Asia, home visits by trained CHWs to promote preventive care as well as to provide curative care reduced neonatal mortality by about 30% (10). However, there are little data on experiences of integrating CHWs into health systems to improve maternal and newborn outcomes in settings in sub-Saharan Africa.

Knowledge gaps exist in defining how to sustainably implement and scale-up community-based interventions for maternal, newborn, and child health, and how these interventions not only improve home care practices but also create demand for healthcare services in the broader health system. The Uganda Newborn Study (UNEST) was among the first community-based trials in sub-Saharan Africa to test a package of home visits during pregnancy and the postnatal period using CHWs linked to health system strengthening for maternal and newborn care (11, 12).

This paper is the third in a series on the impact and findings of UNEST. This qualitative study sought to document experiences within UNEST of CHWs in promoting healthy home behaviours and demand for maternal and newborn care services, to identify community perceptions of this CHW role, and to identify and explore the factors that influence the performance of CHWs in both urban and rural settings in order to inform future programming.

## Methods

### Setting

This study was carried out in the Iganga/Mayuge Health Demographic Surveillance Site (HDSS), located in Iganga and Mayuge districts in south-eastern Uganda. The HDSS covers an area of 155 km^2^ and has a population of about 70,000 people, at the time of the study, in 65 villages, 18 parishes, and 12,000 households. It is a largely rural area, with the main economic activity being subsistence farming. Within the HDSS, there is one government hospital, 15 health centres, 24 private clinics, and other informal health providers, mainly traditional birth attendants and drug shops. These informal health providers are mainly found in small trading centres.

### Intervention

The UNEST package was developed through formative research which included a literature review, consultation with technical experts, policy makers, local leaders, and community-based data collection to inform the intervention design and development of counselling and behaviour change materials, the details of which have been described elsewhere (12). Sustainability and scale-up feasibility were considerations from the outset of the study design phase. The intervention delivered in the cluster randomised control trial comprised a community-based home visit package which linked to health facility services. CHWs were recruited from the community (Box 1), based on criteria stipulated in the national Village Health Team guidelines: a regular/permanent resident of the community, literate and willing to work as a volunteer (13). A preference was given to mature females already doing some community health work. They were trained to identify pregnant women and make two pregnancy visits and three postnatal visits in the first week after birth, providing health education on pregnancy, preparation for childbirth and newborn care (Box 2). More details of this study can be found in the published UNEST protocol paper (12).

Box 1 **CHW selection, training and deployment**

**Selection process**

- CHW post advertised according to criteria set using Ministry of Health Village Health Team criteria
- Community identifies a selection team
- Candidates interviewed and CHW post filled

**CHW training**

- Five-day training on preventive and promotive maternal and newborn care and counselling skills
- Teaching methods include participatory discussion, practical demonstrations and role-playing

**CHW deployment and supervision**

- CHWs were given a set of materials including a register, picture-based counselling cards on birth preparedness and maternal and newborn care, an example of the ‘mama kit’ (delivery kit) and monthly reporting forms
- For easy identification they were given branded t-shirts and identity cards
- Directly observed supervision visits and group supervision meetings took place monthly until competency was reached, and quarterly thereafter
- Supervisors were nurses/midwives from the local health unit
- ‘Super’ CHWs nominated as leaders were tasked with mobilising and encouraging fellow CHWs and serving as supervisors where health workers were not available
- CHWs were instructed to identify pregnant women in their catchment area, make two home visits during pregnancy and three home visits in the first week after delivery with extra visits for women or newborns with complications

Box 2 **Content of care during CHW home visits**

***Home visits during pregnancy***

*First pregnancy visit (Target: as early as possible)*

- Counsel on and refer for ANC including tetanus immunisation and malaria prevention
- Counsel on birth preparedness and clean delivery practices
- Counsel on and assess danger signs of pregnancy, refer if present
- Counsel on and refer for HIV testing

*Second pregnancy visit (Target: in third trimester)*

- Counsel on birth preparedness and clean delivery practices
- Counsel on and assess danger signs of pregnancy, refer if present
- Counsel on newborn care practices immediately following delivery (e.g. placing the baby skin-to-skin, ensuring warmth, initiating breastfeeding, hygienic cord care)
- Counsel on newborn danger signs

***Home visits in the postnatal period***

*First postnatal visit (Target: birth day to day 3)*

- Counsel on and assess maternal and newborn danger signs, refer if present
- Promote thermal care (skin-to-skin placement, delayed bathing, and wrapping)
- Support for exclusive breastfeeding
- Encourage cleanliness especially cord care

*Second postnatal visit (Target: day 5–7 after birth)*

- Counsel on and assess maternal and newborn danger signs, refer if present
- Refer for immunisation
- Counsel mother on breastfeeding and postnatal family planning
- Reinforce need to seek care or call CHW for support

*Extra care for sick and/or very small newborns*

- Follow-up visit post-referral, or two extra home visits if referral is not accepted
- Extra counselling on thermal care
- Extra support for breastfeeding
- Extra attention to hygiene

### Data collection

Data were collected through in-depth interviews (IDIs) and focus group discussions (FGDs). Participants included facility-based health workers; members of the District Health Team and local council (village leaders); recipients of CHW services (mothers with children less than 6 months of age); and CHWs selected from both urban and rural areas (Table 1). Interview guides and data collection tools were pretested for each group of respondents and standardised. Key themes explored included the perceptions of CHWs amongst community members, and the experiences of CHWs in carrying out their various roles as well as the facilitators, barriers, and achievements they encountered through their work.

**Table 1:** Overview of study participants and key themes explored

Type of participant	Role in the intervention	Number interviewed	Data collection method	Focus of data collection
District Health Management Team	Selection of CHWs, participate in training and group supervision	2	IDI	Experiences interacting with CHWs Perceptions of technical competency, ability to make linkages and support, continuity of services; effectiveness of CWHs’ work; sustainability, barriers and suggestions to overcome barriers to CHW approach Community behaviours around pregnancy and newborn care
Facility-based health workers (nurses and midwives)	CHW supervisors	6	IDI
Local community leaders (urban and rural)	Sensitisation of communities to CHWs	6	IDI
Community health workers (CHWs)	Home visitors		IDI, FGD	Roles, facilitating factors, achievements, challenges, motivations, expectations, changes associated with their work, experience with health workers
Urban		14	
Rural		18	
Mothers of children under 6 months	Clients of CHWs		IDI	Experiences interacting with CHWs Experience of danger signs and response
Seen at least once by CHW		4	
Seen by CHW and accepted referral		4		

### Data analysis

Interview data were transcribed and analysed using manifest content analysis (14). Field notes, contact summary sheets and transcripts of the tape-recorded interviews and FGDs were read to identify key themes. Pattern coding was done by conducting close and repeated readings of the field notes and contact summary sheets in order to discover patterns within the emerging themes, so as to deduce smaller analytical units to further elaborate the themes. Comparison was made of data from the different participants to explore commonalities of experiences and observations in view of the emerging themes. Results were presented thematically.

Ethical clearance was obtained from Makerere University School of Public Health Higher Degrees Research and Ethics Committee and the Uganda National Council of Science and Technology. In addition, approval was sought from the district authorities and local leaders in the communities where the study is being conducted. Informed verbal and written consent was sought from all study respondents.

## Results

### CHW selection, training, deployment and supervision

While women were the preferred candidates for the CHW role, in practice a number of communities selected men as CHWs. Participants reported that indeed most of the active CHWs and all the CHW leaders (‘super CHWs’) selected by the CHWs themselves were men. CHWs were trained near their homes in a mix of English and the local language, mainly using what was described as a ‘hands on’ approach. The CHWs were also deployed immediately after training and were introduced to the community by the local leaders.

Given the short (7-day) training and immediate deployment, a number of CHW participants reported that they felt ‘not ready’ and needed more supervision than they received. The main challenges to CHW confidence reported by health providers and other community stakeholders were perceived low levels of education and social status of the CHWs in the community, which affected their acceptability at the start of the intervention. At the outset, CHWs were seen as ‘just local people’ who are ‘ordinary’, without any medical training. Said one rural female CHW: ‘Local people used to say we are just from the same village, what do we have to talk to them about?’

Discussions with health workers, community leaders, and CHWs all pointed to the social status of CHWs as a source of tension in the delivery of their services. The CHWs lacked confidence in approaching households of people they considered to be of ‘higher social status’, such as those who were wealthier or more educated, including the families of health workers, sometimes leading to limited or no interaction at all:

When you look at the scenario, it is a bit complicated … it is just like a family talk or a friendly talk as CHWs disseminate information, and the barriers could be the condition they are working in may not be favourable; some families may seem higher than the volunteer, so they may look at the information they are giving as not worth it as regards to the social status or as regards to education status. The poor young man or women feels threatened; yet the project targets the whole community. So, that is a major barrier. (Member, District Health Team)

However, CHWs reported that given time this barrier was overcome with supervision and increased confidence. In UNEST, support supervision was provided first through monthly directly observed supervision visits with each CHW separately and monthly group meetings. The monthly group supervision was later shifted to a quarterly basis once CHWs were considered to be performing adequately in their role. Community members gradually gained trust in the CHW work and began to open up and seek CHWs’ assistance:

As I told you, at first the community members used not to value the CHWs; these days it is the community that explains to those who are hesitant to get the service from us, our importance as CHWs has improved and people who have sick children also come to seek advice from us, which was not the case before. (Male CHW, urban)

At first the people were [*looking down on*] us since we were staying in the same community … they say ‘this person who trained for one week’, but now they appreciate. You would reach a home and they ignore you and they tell you to leave the place, and they tell you as they don't have time to attend to you, but these days they have changed. I think they learnt the importance slowly by slowly. And those we have given referrals so far have appreciated our work and they have done a very big job of sensitising the rest in the communities. Because at first I had a lady whom I used to visit … and she told me she never wanted me to visit her. But later during the course of her pregnancy she got complications with the pregnancy and her legs got swollen … I referred her to Nakavule and they treated her. Since then she knows the importance of CHWs and whenever she could get complications with her body she could come and seek advice from me. (Male CHW, urban)

### Changing beliefs and practices and around pregnancy and newborn care

Pregnancy, childbirth and the newborn period is surrounded by many cultural beliefs and traditional practices that could serve as a barrier to CHW work. Despite this challenge, CHWs were perceived as successful in changing certain practices during pregnancy and for newborn care (Table 2). CHWs revealed that initially it was very difficult for them to identify pregnant women easily, partly due to the local Kisoga culture which dictates that pregnancy be kept a secret. Both health workers and CHWs reported that more women were attending ANC earlier in their pregnancies, with the exception of younger women and older women who were more likely to keep the pregnancy hidden for longer.

**Table 2:** Changes attributed to CHWs’ activities

Change described	Examples given of CHW-enforced behaviours associated with the change
Changes during pregnancy and labour	
Increased ANC attendance	Pregnant women and husbands/partners informing CHWs of a pregnancy; more women attending ANC during the first trimester
Increase in birth- preparedness activities	Husbands/partners save money, provide women with money for emergencies, transport, and babies’ needs
	Women buy mama kit items
	Women ask CHWs to educate them in presence of husbands
Increased knowledge on pregnancy health issues	Women attend to their health needs during pregnancy
	Women try to eat healthy/balanced diets
More likely to seek care	Women recognise danger signs
	Women initiate contact with CHW or health facility in case of swelling, general weakness, or if a decrease in baby's movements felt
	More deliveries at health facilities
Improved health worker/client relationship	Women experience a caring attitude from health workers, as the latter are less constrained when women have mama kits and ANC cards
Health workers recognise referrals	Women with CHW referral slips are seen faster at hospital or health unit
Changes during postnatal period	
Increased awareness of needs of newly delivered mothers and babies	Women put only salty water on the baby's umbilical cord rather than animal dung and herbs
	Bathing is delayed instead of immediately practised
	Men also acknowledge the importance of these practices
	CHWs are sought outside of routine visits regarding danger signs
More use of health facilities	More women taking their newborn babies for postnatal care, including immunisation
	Seeking medical care from qualified persons
	Newly delivered mothers reaching out to CHWs when self or infant is unwell
Improved breastfeeding	Immediate breastfeeding at birth and continuous breastfeeding
	Delayed introduction of other feeds
	More women giving colostrum

CHWs capitalised on social networks to identify pregnant women who would become new clients, learn about births and disseminate information. Husbands were reported to approach male CHWs in particular when their wives fell pregnant. Some women reported getting to know the CHWs through social ties including extended family members and friends. It was further reported that even when fetching water or during social gatherings, community members would inform CHWs about a women with signs of pregnancy, or to report that a woman was in labour. Participants associated several changes to birth preparations in response to the CHW intervention. In particular, the role of men in supporting their expectant wives was mentioned. When the husband was part of counselling during pregnancy, decision making around saving money and seeking care was perceived to be easier:

There is a very big change because now women buy things to use in a health facility, and when you go back to them for the second visit and ask these women to show you the things she can show you, the things you told her to buy. (Male CHW, urban)

I think maternal mortality has reduced because as you know if a mother comes and she doesn't have anything, at times the health workers may not take any interest to handle her, but if she has everything then the care is also given, because she has everything she needs to examine her – she has the gloves, she has everything. The health workers definitely doesn't have a second thought of where to get the gloves or now where will I get the polythene sheet? He or she will examine the woman and attend to her. (Health worker)

Men generally used not to take it serious but now they prepare, and because we teach them before delivery they save or work hard so that they can buy what is necessary during delivery. (Female CHW, rural)

CHWs revealed that it was helpful to start introducing newborn care concepts during pregnancy visits rather than waiting until the baby had arrived. Mothers noted that they learned things about caring for the baby that they didn't know previously, including newborn danger signs and the urgency around seeking care for them. Breastfeeding messages were well received, and support for attachment and positioning was appreciated by mothers. CHWs and health workers reported that practices were more likely to be sustained if supported culturally, regardless of what healthcare staff were promoting.

Participants noted that while communities appreciate the education received, some of the suggested behaviours have been slow to take root. In particular, practices around cord care were difficult to change completely. Women in this study reported that previously they used powder, ash and dung on the baby's umbilical cord in order to make it heal faster. While practices shifted away from placing these types of substances on the cord, the message to place nothing on the cord was not prioritised by CHWs, and even when delivered it was not well received by mothers. Even health workers promoted cleaning the cord with plain water: ‘What has changed is the fact that before, people would apply all sorts of things to the cord, but right now we are told not to apply anything on the cord apart from cleaning it with water’ (Mother, rural).

Changes in behaviour around thermal care were also associated with CHW visits. Women, especially first-time mothers, reported that they were taught how to wrap babies for warmth and noted the importance of delaying bathing and not keeping the babies in wet bedding. However, not all of the targeted practices were equally accepted: skin-to-skin care was not frequently mentioned as a thermal care practice, but rather CHWs and mothers revealed that advice was given to buy cotton clothes, baby hats, and socks to keep the baby warm. However, the few CHWs and mothers who had promoted and practised skin-to-skin care for low-birthweight babies were animated proponents of the practice, having witnessed its dramatic results in babies previously thought to be beyond assistance.

### Referral and linking with health facilities

CHWs revealed that during each routine visit they screen women and newborns for danger signs and refer if needed, sending the mother to a health facility with a referral note so that the health workers see them quickly. Overall health workers considered CHWs to be technically strong in providing knowledge and skills to the families and providing services in accordance with client expectations. Follow-up and feedback were regarded by mothers who had been referred as very important in creating accountability for referral compliance and instilling confidence in the service. Participants including District Health Team members noted that when a health facility is poorly staffed and equipped, there is less motivation for women to comply with referral:

I sent there a mother for delivery, but reaching [the health unit] it was around 5 pm; she couldn't receive the services … She had to go to another health facility and she later came up to [the district hospital]. (Female CHW, rural)

So, the CHW sends a mother and she doesn't receive the services and she comes back; the next time you try to send her to the health facility, it creates a barrier. (Member, District Health Team)

### Barriers and challenges experienced by CHWs

CHWs were faced with a number of challenges that influenced their performance (Table 3). At the operational level CHWs were challenged by a lack of transport, particularly for those with larger areas to cover, which affected their ability to reach their clients at targeted times and in the case of complications. They also revealed that when they propose the use of the ‘mama kit’, families always ask where they could buy the recommended materials because they are not always available. UNEST was implemented in an area where communities are exposed to other health-related research activities. These interventions, which also use community members as volunteers, have different implementation frameworks and support mechanisms for volunteers and ways of engaging communities. As a result, CHWs were faced with demands for material support from families which had received such support from previous studies.

**Table 3:** Overview of challenges faced by CHWs and proposed solutions

Challenge raised	Implications for the intervention	Proposed solution
Transport	Lack of transport, translating into delays in reaching their clients, or not reaching them at all	Request for provision of transport or transport allowances
Mama kits not available	Some pregnant women expect CHWs to provide mama kits	Ensure mama kits are available and in designated clinics and drug shops and subsidised where possible
Confusion about CHW services Research fatigue	An earlier intervention study included the provision of soap and other things to mothers. This created expectations of the same from subsequent home-based interventions.	Encourage harmonised approaches to community activities and for all activities to follow District Health Team protocol
Research fatigue	Given the high level of studies in the intervention area, some women ignored CHWs, citing overuse	HDSS sites need to ensure that they exclude households from multiple or back-to-back studies
Apprehension from some local leaders	Some local leaders felt threatened by the recruitment of volunteers not directly under their supervision	Build trust and establish good rapport with key influentials
Lack of CHW confidence	Few women sought CHW services early on; delayed appreciation for CHW services	Engage local leaders early on in mobilisation and mediation roles as well as community sensitisation Use community structures to clarify roles Develop strong supervision from the outset Provide refresher training and additional opportunities for CHWs to gain skills
Competing demands on CHW time	Target time for home visits not met; clients with danger signs may not approach the CHW with danger signs because the CHW may not be available at the required time	Group counselling during pregnancy with follow-on visits by those who require additional attention Provide incentives for on-time visits Improve links with health workers and local health units
Low CHW uptake, particularly in urban areas and among younger CHW	Women are more educated with more competing demands on their time; CHWs do not feel as welcomed	Increase recognition of CHWs by local leaders in community fora to build confidence in their services Advertise CHW services on the radio and through other media
Cultural barriers around early disclosure of pregnancy	Delayed service delivery	Continued community sensitisation around the importance of early care-seeking Map social networks and identify key informants
Poor linkage between CHWs and local health facility	Women arrive for care when facilities are closed	Improve links between CHWs and local health facilities Ensure CHWs know the services and the times at which these services are available at the facilities

CHWs indicated that at the beginning they were received with mixed feelings by local leaders, who felt threatened. This is despite the fact that the local leaders were involved in the mobilisation for the selection of CHWs. In addition, CHWs were initially viewed as fellow community members with limited expertise to teach about health matters. Specific households in the community regarded as having high status, such as politicians, rich households, and homes of health personnel, were avoided with the thought that they did not really need their service. On a personal level CHWs admitted that despite their desire to fully serve the communities, they also have personal demands on their time, particularly the CHWs in urban areas.

The top performance motivator noted by CHWs was financial benefit, which in the UNEST intervention was limited to a small stipend linked to supervision visits. Other factors mentioned were improved social status (including being called *musawo*, or doctor), a sense of civic responsibility, material benefits such as the kit and t-shirt, and the supervision received. Some of the CHWs saw volunteering as a temporary alternative to unemployment. It offered them an opportunity to put their skills to use in an area where they are needed.

## Discussion

This qualitative study nested in one of the first trials to evaluate home visits during pregnancy and the newborn period in sub-Saharan Africa reveals shifts in the way community members and health workers perceive CHWs within a routine health system structure. Over a short period of time, a new cadre of mostly male CHW was accepted and able to navigate long-held beliefs and practices relating to maternal and newborn care. However, to be successful certain conditions must be met during their selection, training, deployment, supervision, and motivation, in order to overcome the barriers experienced and result in a sustainable, effective community-based service.

Overall social influence, trust, and culture had a strong bearing on community adoption of the behaviours and practices promoted by CHWs. Once trust was established, pregnant women and their families were willing to listen to the CHWs and to respond to referral. A key component to establishing this trust was ensuring male involvement. This was most pronounced in terms of birth preparedness, as men still dominate economic power and related decision making in many households in Uganda (15).

Social influence on health behaviour is a recurrent theme in health promotion literature. This includes the importance of engaging the various social structures that exist in the community, like traditional leadership structures, women's groups, and faith-based groups. Wide engagement has the potential to broaden the ownership of the programme and gain extra support for the CHWs as well as for families (16, 17). The importance of community connections to identify pregnant women and new deliveries suggests that social networks could be strategically mapped and utilised to reach audiences and to disseminate messages. The lack of success in identifying pregnancies among high-risk groups of older and younger women points to the need for CHWs to not only rely on house to house visits, but on other forms of community mobilisation and awareness on services available for families expecting a newborn.

While CHWs were for the most part able to deliver the messages, they learned during training, some of the messages were not accepted as readily as others. Completely changing practices around umbilical cord care, for example, was a challenge. Formative research is critical to identifying the local understanding of chosen messages and barriers to practising uptake. Interventions such as kangaroo mother care for preterm babies, while practised by few participants, generated enthusiastic converts even amongst mothers with normal weight babies and points to an opportunity to use mothers as peer counsellors and champions. Identifying newborns with danger signs and ensuring prompt care-seeking was challenged by the routine visit schedule (i.e. passive case finding). Compliance with referral was thought to be high but also associated with the perceived quality of care at the nearest health facility.

The way CHWs are selected, trained, managed, and supported is central to the quality of services that they deliver (18). Community involvement in the selection process was considered important in CHWs being accepted, a finding confirmed by several other studies (9, 19, 20). However, in urban areas it was difficult to achieve widespread community involvement. Involving local stakeholders in training was also used to ensure buy-in and accountability for the quality and content of the training. The length of training was quite short given the scope of the CHWs’ responsibilities, resulting in a greater emphasis being placed on routine supervision. The immediate deployment of CHWs following training resulted in greater retention of messages, but also meant that CHWs felt unprepared. Initially scheduling monthly supervision was sufficient, until demand for services increased and the CHWs were confident in their role. The use of existing government health workers as supervisors resulted in technically strong support, in addition to creating an additional link between the community and health facility. However, facility-based health workers were not always available, and in some cases motivated CHWs served as peer supervisors. The lack of refresher trainings was compensated for by on-the-job training and mentorship, and this was effective in ensuring knowledge and skill retention (21). If CHWs were included as a permanent feature in the health services, there would be more experienced CHWs who could mentor the newly trained ones for a few weeks, so that when they start in their own communities they have more confidence.

While CHWs noted that financial incentives were key to job performance, intrinsic motivation and job satisfaction were also driving forces. They considered appreciation from community members and the supervision they received from formal health workers as important motivators. Several studies in Nepal and other parts of Africa (Gambia, Ethiopia) show that community acknowledgement is a critical motivation (19, 22, 23). Studies from Tanzania and South Africa also found that money was the top motivator while non-monetary incentives act as enablers (24). A recent meta-analysis on lay health workers found financial compensation to be complicated; some unsalaried lay health workers wanted regular payment, while others were concerned that payment might threaten their social status or lead recipients to question their motives (25). One concern with the volunteer-based position is that some CHWs viewed their role as a temporary alternative to being unemployed. While retention was not a major problem in this study, if run over a longer period of time or scaled up more widely it might pose a problem for CHW reliability.

There are some limitations to this study. Our assessment relied on knowledge, attitudes, and perceptions of CHWs, mothers, health workers, and key stakeholders pertaining to the UNEST intervention. The content of care provided by CHWs was not independently verified through observation of a home visit, although CHW competency is reported elsewhere (21). The CHWs were those who were still active at the end of the implementation phase, so there is no information from CHWs who left their posts. In addition, the caregivers interviewed were limited to those with live children, because of the additional sensitivity involved in talking to parents whose children had died. Despite these limitations, this study examined the implementation experience and identified areas for strengthening the maternal and newborn component of CHW programmes that can be applied in Uganda and similar settings.

This study raises several questions for additional research. Given issues around CHW confidence, the optimal duration of training and intervals for refresher training and/or on-the-job mentorship needs to be further established. The specific challenges faced by CHWs working in urban areas are also an area of very little research, with implications for successful programme scale-up. A major factor that will affect the scale-up of UNEST is the capacity of districts to integrate CHWs into routine systems. In the case of UNEST, district authorities and health workers at health facilities were involved in all aspects of the project, from design to implementation, evaluation, and dissemination. However, owing to limited fiscal and decision space, districts currently do not have additional resources to effectively support, scale-up, and sustain the initiative. Future programmes should explore innovative mechanisms to achieve this, including ensuring that all activities are planned and budgeted for in annual district plans.

## Conclusion

There is potential in the role of CHWs in improving maternal and newborn care in Uganda. However, selection of these workers is a sensitive process that requires a tailored approach for urban compared to rural areas and community involvement in order to foster trust, support, and acceptability. It is essential that CHW training includes problem-solving skills and guidance on integrating technical knowledge with cultural sensitivities, so that service delivery is context-specific. Strengthening links between facility-based health workers and CHWs as well as improving quality of facility care cannot be overlooked.
